# Effects of a 12-week dance program with two weekly frequency protocols on fine motor competence and balance in preschool children: A randomized controlled trial

**DOI:** 10.1371/journal.pone.0338422

**Published:** 2025-12-09

**Authors:** Anđela Đošić, Danijela Živković, Slavoljub Uzunović, Mladen Živković, Nenad Đorđević, Saša Pantelić

**Affiliations:** 1 Faculty of Sport and Physical Education, University of Niš, Serbia; 2 Councilor for Sports, City Council, Vranje, Serbia; Universitatea Transilvania din Brasov, ROMANIA

## Abstract

This study examined the effects of structured dance programs on motor competence in preschool children. In a 12-week randomized controlled trial, 80 children were randomly assigned to two dance intervention groups (EG1: 2 × 35 min/week; EG2: 3 × 25 min/week) or a control group (CG) following the standard physical education curriculum. Fine motor skills, fine motor integration, and balance were assessed using the Bruininks–Oseretsky Test of Motor Proficiency, administered both before and after the intervention. Significant improvements were observed in EG1 for fine motor precision (p < 0.001), fine motor integration (p = 0.022), and static balance (standing on one leg on a balance beam with eyes open; p < 0.001). EG2 showed significant gains in dynamic balance (walking forward on a line; p < 0.001). Both dance programs enhanced preschoolers’ motor competence compared to the control, with higher session volume producing superior outcomes. These results support integrating structured dance sessions into preschool curricula to effectively enhance motor competence, offering a practical strategy to promote physical development in early childhood.

## Introduction

The preschool period is when the body is most adaptable to external influences, especially physical activity [1]. During this time, children develop basic motor skills such as walking, running, jumping, throwing, and catching [2,3]. Because of this rapid development, parents and caregivers should encourage these activities to support proper motor growth [4]. This stage is crucial for developing coordination, strength, and other motor habits that affect overall health and well-being [5,6]. Although individual differences exist, preschool age remains the optimal period for motor development [7]. Studies highlight that developing motor skills and competence is crucial for lifelong participation in sports and physical activity [8]. Children with high levels of motor competence develop better cognitive abilities [9], cardiorespiratory fitness [10], have reduced levels of hyperactivity [11], and increased future academic achievements [12].

Preschool years provide an ideal opportunity for children to develop lasting habits and preferences for physical activity [13]. Telama et al. [14] found that children who are active in early childhood tend to remain active into adulthood. Despite the well-documented benefits of physical activity [15], research indicates a decline in activity levels among preschoolers [16–18], with four- and five-year-olds being less active than three-year-olds [19]. Overall physical activity also decreases with age [19]. Current WHO guidelines for physical activity in children aged 5–17 recommend 60 minutes of moderate to vigorous physical activity daily [20]. However, evidence shows that a large number of preschool children do not meet these recommendations [16–18]. The authors of the review study Barbosa and Oliveira [21] found that children in preschool institutions spend most of their time sedentary, highlighting the need to adapt play areas in ways that promote greater physical activity. Insufficient physical activity among preschool children in kindergartens in the territory of the Republic of Serbia has shown an increase in the number of children with flat feet and fallen arches from generation to generation, prompting initiatives for projects aimed at increasing physical activitie [22]. There is a cause-and-effect relationship between the level of physical activity and motor competence namely, children who are more physically active will have higher levels of motor abilities [23], and vice versa [24].

Children’s motor abilities can be enhanced through various structured exercise programs [25,26], whose effectiveness depends on program duration, intensity, and frequency. Plazibat et al. [27] implemented a 10-month program consisting of gymnastics and running, conducted three times per week for 60 minutes, and reported significant improvements in most motor abilities. Similarly, Zhang et al. [28] reported that an 11-week aerobic program (four sessions per week, 40 minutes each) enhanced cardiorespiratory and muscular fitness, as well as speed and flexibility. In contrast, Thomaidou et al. [29] found that a shorter, two-month program (two sessions per week, 45 minutes each) improved creativity but not motor skills. Recent systematic review [30] indicate that programs lasting less than 8 weeks are not sufficiently effective in improving motor competence.

The aforementioned programs show more or less positive effects on certain motor abilities of preschoolers. The issue that needs to be addressed, though, is what kind of physical activity is best suitable and acceptable for preschoolers. Also, there is uncertainty about the duration of one training session, the frequency needed during the week, and how long the overall program should last to achieve effects on the gross and fine motor skills of preschool children [29]. However, the existing body of research provides limited evidence regarding the effects of a combined protocol that integrateselements of traditional and children’s dance on the development of motor competence and engagement in early physical education among preschoolers.

Dance programs provide an exceptional opportunity for engaging preschool children in physical activity. Dance encompasses a wide range of movements, coordination, strength, and endurance, and according to research, dance is the second most popular activity among school children, right after soccer [31]. It is based on basic elements of classical ballet, choreography, and gymnastic skills, allowing children to adopt and develop basic motor skills, knowledge, and abilities [32]. Unlike traditional sports, dance promotes active participation and fosters body awareness without the pressures associated with competition [33]. Previous studies highlight dance as a highly significant type of physical activity, giving it an edge over some other types of physical activities [33]. Dance contributes to the development and improvement of motor skills [34–37], motor creativity [28], and its effects have been identified in the prevention and correction of postural deformities [36].

The authors note that the impact of dance on preschool children’s motor competence remains insufficiently explored, requiring more comparative data with other physical activities [28,36]. Previous studies have mainly examined single dance types—traditional or children’s—raising the question of how a combined dance program might influence motor competence compared with the standard physical activity programs recommended by the Ministry of Education and Sports. Based on the above the aim of the study was to determine whether different dance program models lasting 12 weeks (with varying frequencies) are more effective compared to the regular physical activity program recommended by the Ministry of Education and Sports and whether they influence changes in the motor competence of preschool children. We hypothesize that dance programs will have greater effects and enhance the motor competence of preschool children compared to children involved in the regular physical activity program.

## Materials and methods

### Participants

We included eighty children aged 6.46 ± 0.37 years (42 boys and 38 girls) in the study. We randomly selected participants from children whose parents provided informed consent. Because we recruited participants from three different preschool institutions, we conducted randomization within each institution to ensure balanced group allocation and to minimize potential clustering effects. Sex distribution was not predetermined and occurred naturally, without researcher influence. Minor variations in the number of boys and girls per group resulted from independent randomization procedures conducted within each participating preschool.

We applied the following inclusion criteria: preschool children aged 6.5 years ± 6 months; absence of chronic diseases that could affect testing outcomes; no history of locomotor system surgeries; absence of respiratory dysfunctions; no visual or hearing impairments (as verified by each child’s medical record submitted to the kindergarten registry); no participation in other organized forms of physical activity apart from those conducted within the preschool institution; and parental or guardian consent for participation in the study.

We applied the following exclusion criteria: children with developmental disorders; children who were recovering from injuries or illnesses; children who were receiving any form of pharmacological therapy; children with motor system dysfunctions affecting balance; and children whose parents or guardians did not provide consent.

To determine the sample size, we used G*Power 3 [38] with an effect size of f = 0.40, alpha of 0.05, and power of 0.80. An effect size of f = 0.40 was selected based on previous similar studies (Jouira et al., 2024) that reported medium to large effects, representing the expected magnitude of impact. We applied a statistical power of 0.80 to ensure adequate sensitivity for detecting true effects while maintaining control over Type I error.

**Fig 1:**
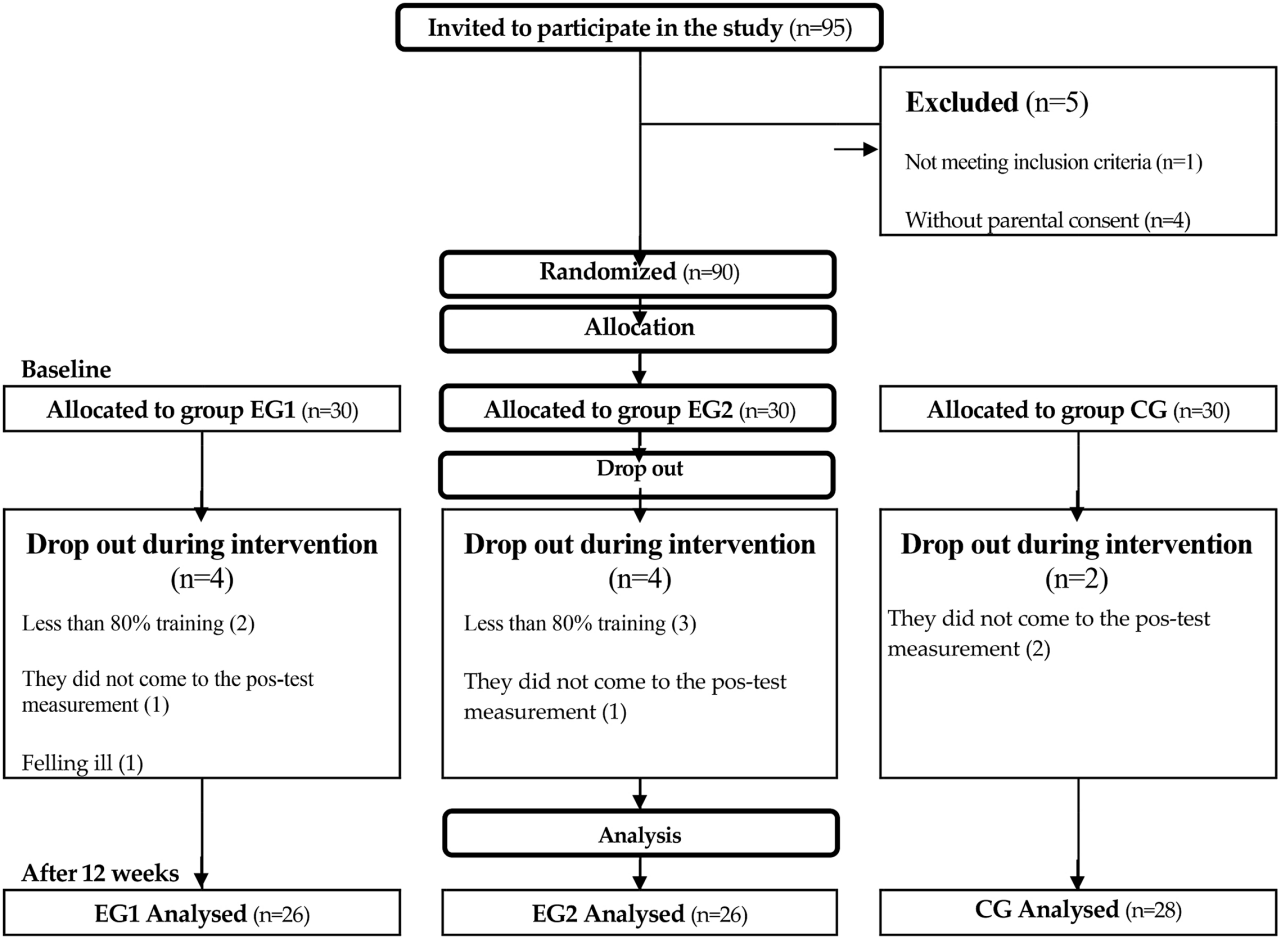
Flow chart of subjects involved in research, randomization and analysis, including EG1-2x35min dance, EG2 3x25min dance, CG- 3x25min Physical Education.

Based on the aforementioned assumptions, we determined that the desired sample size was 73. We present a detailed overview of participant selection in Fig 1. Out of the initial 90 children, 10 did not complete the program. The reasons for withdrawal were participation in fewer than 80% of the training sessions (5 children), absence from the final assessment (4 children), and illness (1 child). The remaining participants fully completed both the intervention and the post-testing procedures. The total sample at the final measurement consisted of two experimental groups (dance groups, EG1 and EG2) with 26 children each, and one control group of 28 children (CG).

The children attended one of the 3 preschool institutions included in the experiment, for 9–10 hours per day, five days a week [39], within which they had the following activities: breakfast and indoor activities, outdoor activities, nap time, lunch, and free play.

### Study design

We tested the motor competence of the children before and after the 12-week program. Before starting the experimental protocol, we informed parents and guardians about the procedures and possible risks, and each parent or guardian provided consent for their child to participate voluntarily in the study. We conducted the research in accordance with the principles of the Helsinki Declaration and recommendations for research involving human participants [40]. The study was approved by the Ethics Committee of the Preschool Institution “Naše dete” (No. 1675-07/17), date of approval December 13, 2017.

Parents and guardians attended both the initial and final testing sessions. The Directorate for Preschool Education and Upbringing reviewed and approved all experimental procedures and study materials.

We implemented all programs in physical education halls at the same time of day and with the same instructors. We carried out all measurements in the morning hours, between 9:00 and 11:30 a.m., to control for potential diurnal variations in performance. We provided rest intervals of approximately 2–3 minutes between tests to prevent fatigue.We conducted the initial testing prior to implementing the experimental programs, over the course of two days, following the standardized sequence recommended in the official testing manual. On the first day, we administered tests assessing fine motor skills (Drawing Lines Through Paths; Folding Paper), fine motor integration (Copying a Square; Copying a Star), manual dexterity (Transferring Pennies), and bilateral coordination (Jumping in Place—Same Sides Synchronized; Tapping Feet and Fingers). On the second day, we administered tests of balance (Walking Forward on a Line; Standing on One Leg on a Balance Beam—Eyes Open), running speed and agility (One-Legged Stationary Hop), upper-limb coordination (Dropping and Catching a Ball—Both Hands; Dribbling a Ball—Alternating Hands), and strength (Push-Ups; Sit-Ups).

We applied the same testing protocol during the post-intervention assessment. All equipment was calibrated according to the manufacturer’s instructions prior to each testing session. Two instructors, both physical education teachers who had completed specialized training for the program Playing, Singing, Creating through Dance, implemented the experimental programs. Both instructors participated simultaneously in delivering all sessions to minimize potential instructor bias. We conducted the intervention period from September 1 to November 30, 2018. Experimental Group 1 (EG1) performed the program on Tuesdays and Thursdays, while Experimental Group 2 (EG2) performed the program on Mondays, Wednesdays, and Fridays. The Control Group (CG) completed its program on Tuesdays and Thursdays. Two educators who had previously undergone training for program implementation delivered all experimental programs. Qualified staff (professors and physical education and sports teachers) carried out the initial and final testing during the children’s regular attendance in kindergarten. For a child to be included in the data analysis, they had to meet a minimum participation rate of 80% in the experimental program. Children could withdraw from the study at any time, either by their own choice or at the request of their parents or guardians. To ensure children’s privacy, we did not record any video materials during program implementation or testing.

### Measures

#### Anthropometric measurements.

The age and anthropometric measurements of the subjects are shown in Table 1.

**Table 1:** Age and anthropometric measurements of the subjects (mean ± standard deviation).

	EG1 (n = 26)	EG2 (n = 26)	CG (n = 28)
	15 boys and 11 girls	10 boys and 16 girls	17 boys and 11 girls
Age (years)	6.53 ± 0.33	6.47 ± 0.31	6.32 ± 0.40
Body mass (kg)	25.40 ± 6.34	23.61 ± 3.55	25.15 ± 5.60
Body height (cm)	120.84 ± 5.06	121.03 ± 4.48	122.10 ± 4.78
BMI (kg/m^2^)	17.27 ± 3.42	16.07 ± 1.89	16.73 ± 2.69

Legend: BMI, Body Mass Index; EG1, experimental group 1 (2 session/week, 35 min/session, dance); EG2, experimental group 2 (3 session/week, 25 min/session, dance); CG, control group (2 session/week, 35 min/session, standard physical education.

Anthropometric measurements were conducted by trained staff following the recommendations of Eston & Reilly [41] and included measurements of body height and body mass. Body height measurement was conducted using a stadiometer by Martin (GPM, Switzerland) (measurement accuracy 0.5 cm). The Omron BF511 (Omron, Osaka, Japan) scale was used to measure body mass with an accuracy of 0.1 kg. Body Mass Index (BMI) was calculated as the ratio of body mass (kg) to body height (m^2^) [42]. Every anthropometric measurement was carried out by qualified and experienced professionals.

#### Motor competence.

To assess the motor proficiency of children, the short form of the Bruininks-Oseretsky Test of Motor Proficiency, Second Edition (BOT-2 SF) [43] was administered, consisting of 14 tests (Drawing lines through path; Folding paper; Copying a square; Copying a star; Transferring pennies; Jumping in place-same sides synchronized; Tapping feet and fingers; Walking forward on a line; Standing on one leg on a balance beam—eyes open; One legged stationary hop; Dropping and catching a ball—both hands; Dribbling a ball-alternating hands; Push-ups; Sit-ups) that evaluate the fine and gross motor skills of children.

The obtained values in seconds required to complete individual tests were converted into numerical values (points) used as an index for assessing motor competence [44]. The BOT-2 test battery has demonstrated good psychometric properties [44–46]. In our study, the Cronbach’s alpha coefficient was 0.70.

### Physical activity intervention

The experimental dance programs were designed as part of the “Playing, Singing, Creating through Dance” program, accredited by the Institute for the Promotion of Education and Upbringing of the Republic of Serbia, under registration number 965 [47]. The total duration of all programs was 12 weeks (Table 2). EG1 conducted the dance program 2 times per week (total number of sessions = 24), with each session lasting 35 minutes. The same frequency and duration of individual sessions were also applied in the CG officially recommended regular exercise programme) [48]. EG2 conducted the dance program 3 times per week (total number of sessions = 36) with each session lasting 25 minutes. The total training volume for all groups on a weekly basis ranged from 70 to 75 minutes. Each individual session consisted of three phases (with varying durations de-pending on the program): 1) introductory, 2) main part, and 3) final part (Table 2). The introductory phase included natural movements (walking, jogging) to music, stylized walking to specific characteristic music (Pasodoble, Cha-cha), shaping exercises (adapted to the age group) to music, and preschool program games. The main part of the session was conducted following the introductory phase (warm-up), during which children performed either the dance programs of varying frequencies or the officially recommended regular exercise program. Children assigned to the experimental dance groups performed age-appropriate traditional and modern dances. The content was identical across both dance groups, with the only difference being the frequency of practice. In the final part of the session, breathing and relaxation exercises, various games, and activity analysis were performed. The CG also had a three-part structure. In the introductory part, natural forms of spinning were performed. The main part was focused on the development of children’s motor skills, while the secondary part included relaxing exercises. The main part comprised of physical activities conducted through extended and guided play, with an emphasis on free play, as well as simple exercises prescribed by the plan of the Ministry of Education of the Republic of Serbia. The total duration of these physical activities was 70 minutes. According to the Guidebook on the Fundamentals of the Preschool Education Program, the motor skill development plan is applied annually, with activities aimed at fostering the development of all motor skills being applied uniformly throughout the year. The weekly program structure for EG1, EG2, and CG is presented in Table 3.

**Table 2:** Establishment of experimental classes for each group.

Group	Frequency	Duration	Total mins/week	Total training	Part of the class	Duration
EG1	2 х/week	35 minutes	70 minutes	24	Introductory Main part Final	7 minutes 25 minutes 3 minutes
EG2	3 х/week	25 minutes	75 minutes	36	Introductory Main part Final	3 minutes 20 minutes 2 minutes
CG	2 х/week	35 minutes	70 minutes	24	Introductory Main part Final	3 minutes 25 minutes 7 minutes

Legend: EG1, experimental group 1 (2 session/week, 35 min/session, dance); EG2, experimental group 2 (3 session/week, 25 min/session, dance); CG, control group (2 session/week, 35 min/session, standard physical education).

**Table 3:** Structure of the program by weeks for each group.

Weeks	EG1	EG2	CG
Week 1	2 × “Roger Rabbit” children’s dance	3 × “Roger Rabbit” children’s dance	2 × leg strengthening exercises
Week 2	2 × Quadrille	3 × Quadrille	2 × ball games
Week 3	2 × “Jump” children’s dance	3 × “Jump” children’s dance	2 × of hoop activities
Week 4	2 × “Magical journey” 2 (part I)	3 × “Magical journey” 2 (part I)	2 × elastic band exercises
Week 5	2 × “Magical journey” 2 (part II)	3 × “Magical journey” 2 (part II)	2 × agility course
Week 6	2 × “Magical journey” 1 (part I)	3 × Magical journey 1 (part I)	2 × flexibility course
Week 7	2 × “Magical journey” 1 (part II)	3 × “Magical journey” 1 (part II)	2 × arm and shoulder muscle strengthening with equipment
Week 8	2 × “Brankovo kolo” tradicional dance	3 × “Brankovo kolo” tradicional dance	2 × thematic movement games
Week 9	2 × “Spinko” Waltz 2	3 × “Spinko” Waltz 2	2 × cooperative games
Week 10	2 × Folk dance performed in a circle	3 × Folk dance performed in a circle	2 × basic games
Week 11	2 × “Oki Boki Zoki” children’s dance	3 × “Oki Boki Zoki” children’s dance	2 × speed course
Week 12	2 × Boyer	3 × Boyer	2 × strength course

Legend: EG1, experimental group 1 (2 session/week, 35 min/session, dance); EG2, experimental group 2 (3 session/week, 25 min/session, dance); CG, control group (2 session/week, 35 min/session, standard physical education).

The program instructors maintained attendance records for each participant. The overall attendance rate for the program was approximately 90% across all groups.

### Dance styles and setting

The dance patterns consisted of simple movement elements, and the children had no difficulty learning the movements themselves. Their main challenge was remembering the sequence of movements within the dance cycle. Instructors addressed this by increasing the number of repetitions of specific choreography segments and, occasionally, through individual practice. Methodologically, a programmed training approach was applied. All dances were divided into logical units, and only after the previous unit was mastered did the children proceed to the next one.

The program was conducted in a multi-purpose hall used for educational activities, events, workshops, and sports. The floor was made of tarquet, and the hall measured 7 × 8 m (56 m²). The lighting level was maintained at a minimum of 300 lux, and the temperature was set at 21 °C. Ventilation was achieved naturally through window openings, and the relative humidity ranged between 40–60%.

#### Roger rabbit.

The rhythm was set in 4/4 time, with a tempo of 136 beats per minute (bpm). The dance activity was performed to the popular song “Roger Rabbit.” The technical elements included natural walking steps, skipping steps, turning steps around the longitudinal axis, and the “gallop” step. The basic structure was derived from the polka step. It was performed as a partner dance, with participants arranged on the floor in two opposite rows.

#### “Quadrille”.

The dance activity was performed to quadrille music, with a 4/4 rhythm and a tempo starting at 104 bpm and accelerating to 143 bpm toward the end. The choreography was designed for four pairs and represented a ballroom-style dance incorporating elements of stylized walking technique, as well as the cultivation of graceful manners (bows and curtsies) and spatial awareness through frequent partner position changes.

#### “Jump” children’s dance.

The dance activity was performed to an original musical piece, with a 2/4 rhythm and a tempo ranging from 126 bpm to 144 bpm toward the end. In the first part, children performed two skipping steps with a straddle, followed by striking a pose of their choice. In the second part, children performed natural steps in rhythm while moving in a circular formation (progressing along the circle to the left and right).

#### “Magical journey” 2 (part I, part II).

The educational objective of this dance activity was for children to learn the capital cities of the countries from which the featured dances originated, as well as the imaginary modes of transportation used to “travel” to these locations (e.g., airplane, train, bus, ship). The session consisted of a mix of different dances; each associated with a specific country and tempo:

- Introduction: 4/4 rhythm, 120 bpm
- Pre-Vienna: 6/8 rhythm, 157 bpm; Viennese Waltz: 6/8, 60 bpm
- Pre-Madrid: 4/4, 147 bpm; Madrid – Paso Doble: 4/4, 113 bpm
- Pre-Moscow: 4/4, 106 bpm; Moscow – Kazatchok: 4/4, 119 bpm
- Pre-Belgrade: 4/4, 119 bpm; Belgrade – Traditional Dance: 4/4, 118 bpm

#### “Magical journey” 2 (part I, part II).

In the continuation of this educational dance activity, children learned additional dances from other countries. Each segment included a “pre-city” introduction, followed by the dance associated with that location, with corresponding rhythm and tempo:

- Pre-London: 4/4, 115 bpm; London – English Waltz: 6/8, 35 bpm
- Pre-Rio: 4/4, 103 bpm; Rio – Samba: 2/4, 111 bpm
- Greece – Athens (Sirtaki): 4/4, 107 bpm
- Pre-Belgrade: 4/4, 146 bpm; Belgrade – Traditional Dance: 6/8, 56 bpm

#### “Brankovo kolo” tradicional dance.

The dance activity was performed to traditional music, with the first theme set in 4/4 rhythm at 140 bpm and the second theme in a 4/4 rhythm at 70 bpm. The choreography consisted of natural steps performed along a circular path, with participants holding hands while moving to the right and left. Technical elements also included squats and bows as part of the structured sequence.

#### “Spinko” Waltz 2.

The dance activity was performed to an original musical piece, with the first theme in a 4/4 rhythm at 119 bpm and the second theme in a 6/8 rhythm at 59 bpm**.** This activity was designed to teach the rhythm of the waltz**.** The technical elements included steps with foot placement without weight transfer in all directions of movement**.**

#### Folk dance performed in a circle.

The dance activity was performed to an original musical piece in a 4/4 rhythm. Participants performed in a circular formation, holding hands in both directions. The assigned theme was repeated four times, each with varying tempos:

- First repetition: 118 bpm; transition 1: 59 bpm
- Second repetition: 159 bpm; transition 2: 81 bpm
- Third repetition: 197 bpm; transition 3: 97 bpm
- Fourth repetition: 197 bpm

#### “Oki Boki Zoki” children’s dance.

The dance activity was performed to an original popular song with **a** 4/4 rhythm and a tempo of 116 bpm**.** The game incorporated isolated movements of different body parts (e.g., right hand forward – right hand backward) including the arms, legs, hips, and head**.** The activity also included circular movement, performed using both walking steps and skipping steps.

#### “Boyer”.

The dance activity was a traditional ballroom dance performed to traditional music**,** set in a 6/8 rhythm and a tempo of 56 bpm. It was a partnered activity executed in a circular formation**.** Technical elements included stylized walking steps (basse dance), bows, and partner changes within the space**.**

### Statistical analysis

Descriptive data were presented as mean ± standard deviation. One-way ANOVA was used for group comparisons at baseline and post-intervention, followed by LSD Post Hoc test for pairwise comparisons. Differences between the two measurements within groups were determined using Cohen Effect size (ES) [49]. Effect size criteria were as follows: < 0.2 trivial effects, 0.2–0.6 small effects, 0.6–1.2 moderate effects, 1.2–2.0 large effects, and >2.0 very large effects [50]. Univariate analysis of covariance (ANCOVA) (General Linear Model) with eta values was used to determine the effects of experimental programs [51]. Data were analyzed using the Statistical Package for Social Sciences (SPSS) (v18.0, SPSS Inc., Chicago, IL, USA). The level of statistical significance was set at p < 0.05.

## Results

The average session attendance rate was 91% in EG1, 89% in EG2, and 94% in the CG. No injuries or excessive dropouts were recorded during the implementation of the experimental program. The number of participants who dropped out during the implementation of the program is shown in Fig 1. Based on the attendance records, the instructors determined that overall engagement was very high.

In Table 4, the basic parameters of descriptive statistics and differences at baseline and post-intervention are presented. The initial measurement had shown that there had been significant differences between groups at baseline in Copying a square (F = 3.16, p < 0.05), Jumping in place (F = 6.23, p < 0.01), Standing on one leg on a balance beam (F = 3.54, p < 0.01), Dribbling a ball—alternating hands (F = 8.63, p < 0.01), and Push-ups (F = 4.82, p < 0.05). At the final measurement, differences were observed in Drawing lines through path (F = 3.10, p < 0.05), Walking forward on a line (F = 51.32, p < 0.01), and Standing on one leg on a balance beam —eyes open (F = 24.76, p < 0.01).

**Table 4:** Descriptive statistics and differences in motor competence between groups at initial and final measurement (one-way ANOVA + LSD Pos Hoc).

	Baseline				After 12 weeks			
	EG1 (*n *= 26)	EG2 (*n *= 26)	CG (*n *= 28)	Sig	EG1 (*n *= 26)	EG2 (*n *= 26)	CG (*n *= 28)	Sig
Drawing lines through paths	4.88 ± 1.18	5.65 ± 1.33	5.50 ± 1.48	NS	6.69 ± 0.55^**$**^	6.27 ± 1.00	6.04 ± 1.23^**$**^	0.050*
Folding paper	3.81 ± 1.60	3.58 ± 1.94	3.46 ± 1.75	NS	4.27 ± 1.19	4.54 ± 1.92	3.96 ± 1.75	NS
Copying a square	3.31 ± 1.12^**&$**^	3.88 ± 0.86^**&**^	3.86 ± 0.80^**$**^	0.048*	4.35 ± 0.69	4.35 ± 0.63	3.96 ± 0.84	NS
Copying a star	1.54 ± 1.45	1.88 ± 1.66	2.04 ± 1.64	NS	3.58 ± 1.45	3.46 ± 1.70	2.64 ± 1.62	NS
Transferring pennies	5.04 ± 0.87	4.96 ± 1.04	4.86 ± 0.97	NS	5.05 ± 0.92	4.88 ± 1.11	5.25 ± 1.04	NS
Jumping in place-same sides synchronised	2.38 ± 0.50^**$**^	2.35 ± 0.49^**‡**^	1.71 ± 1.15^**$‡**^	0.003**	2.69 ± 0.55	2.73 ± 0.45	2.61 ± 0.69	NS
Tapping feet and fingers	3.42 ± 0.95	3.23 ± 1.11	3.14 ± 1.30	NS	3.92 ± 0.27	3.77 ± 0.51	3.82 ± 0.48	NS
Walking forward on a line	3.73 ± 0.45	3.58 ± 0.81	3.25 ± 0.89	NS	3.85 ± 0.46^**$**^	3.92 ± 0.27^**‡**^	2.50 ± 0.84^**$‡**^	<0.000**
Standing on one leg on a balance beam—eyes open	3.38 ± 0.75^**&$**^	3.15 ± 0.97^**&**^	2.75 ± 0.93^**$**^	0.034*	3.62 ± 0.75^**$**^	3.54 ± 0.65^**‡**^	2.32 ± 0.86^**$‡**^	<0.000**
One-legged stationary hop	7.73 ± 1.25	6.92 ± 1.50	7.68 ± 1.95	NS	8.12 ± 1.11	8.15 ± 1.16	8.07 ± 1.05	NS
Dropping and catching a ball—both hands	4.04 ± 1.31	4.12 ± 1.58	3.54 ± 1.92	NS	4.85 ± 0.3	4.77 ± 0.51	4.57 ± 0.88	NS
Dribbling a ball—alternating hands	3.31 ± 1.32^**&**^	1.69 ± 1.32^**&‡**^	2.75 ± 1.60^**‡**^	0.000**	4.42 ± 1.70	3.58 ± 1.60	3.82 ± 1.91	NS
Push-ups	4.62 ± 0.90^**$**^	4.12 ± 0.91	3.82 ± 1.02^**$**^	0.011*	4.31 ± 1.29	4.08 ± 1.62	4.18 ± 0.86	NS
Sit-ups	3.46 ± 1.24	3.08 ± 0.85	3.68 ± 0.82	NS	3.50 ± 0.99	3.58 ± 0.58	3.79 ± 0.83	NS

Legend: EG1, experimental group 1 (2 session/week, 35 min/session, dance); EG2, experimental group 2 (3 session/week, 25 min/session, dance); CG, control group (2 session/week, 35 min/session, standard physical education); NS, not significant; Sig., the level of significance, ** p < 0.01, * p < 0.05; LSD Post Hoc test between groups - & EG1:EG2, $ EG1:CG; ‡ EG2:CG; The values are ex-pressed as the means ± SD.

Table 5 shows the differences between baseline and post-intervention measurements for each group. Changes after the intervention were observed in most of the variables examined. Among participants in EG1, the largest changes were found in Drawing lines through path (1.96) and Copying a star (1.43), while for EG2, Dribbling a ball—alternating hands (1.28) was the only parameter with large changes. Other effect size values for EG1 and EG2 ranged from small (0.01) to moderate (1.11). For participants in CG, the effect size ranged from small to moderate, with values ranging from 0.12 to 0.94.

**Table 5:** Effect size and Δ(%) between baseline and after 12 weeks.

		Group	
	EG1 (*n* = 26)	EG2 (*n* = 26)	CG (*n* = 28)
	ES (Δ%)	ES (Δ%)	ES (Δ%)
Drawing lines through paths	1,96^d^ (27,06%)	0,52^b^ (9,89%)	0,39^b^ (8,94%)
Folding paper	0,32^b^ (10,77%)	0,49^b^ (21,15%)	0,28^b^ (12,63%)
Copying a square	1,11^c^ (23,91%)	0,62^c^ (10,80%)	0,12^а^ (2,53%)
Copying a star	1,43^d^ (56,98%)	0,94^c^ (45,66%)	0,36^b^ 22,73(%)
Transferring pennies	−0,01^а^ (0,20%)	−0,07^а^ −1,64(%)	0,33^b^ (7,43%)
Jumping in place-same sides synchronised	0,58^b^ (11,52%)	0,04^а^ (13,92%)	0,94^c^ (34,48%)
Tapping feet and fingers	0,70^c^ (12,76%)	0,61^c^ (14,32%)	0,69^c^ (17,80%)
Walking forward on a line	0,26^b^ (3,12%)	0,56^b^ (8,67%)	−0,86^c^ (−31,00%)
Standing on one leg on a balance beam—eyes open	0,32^b^ (6,63%)	0,47^b^ (11,02%)	−0,48^b^ (−18,53%)
One-legged stationary hop	0,32^b^ (4,80%)	0,91^c^ (15,09%)	0,24^b^ (4,83%)
Dropping and catching a ball—both hands	0,84^c^ (16,70%)	0,55^b^ (13,63%)	0,68^c^ (22,54%)
Dribbling a ball—alternating hands	0,72^c^ (25,11%)	1,28^d^ (52,79%)	0,60^c^ (28,01%)
Push-ups	−0,27^b^ (−7,19%)	−0,03^а^ (−0,98%)	0,38^b^ (8,61%)
Sit-ups	0,03^a^ (1,14%)	0,68^c^ (13,97%)	0,13^a^ (2,90%)

Legend: EG1, experimental group 1 (2x/35 min week, dance); EG2, experimental group 2 (3x/25 min week, dance); CG, control group (2x/35 min week, standard physical education); ES, Effect Size - Cohen’s d – a trivial, b small, c moderate; d large; (Δ%), Post-pre change in percentage.

**Fig 2:**
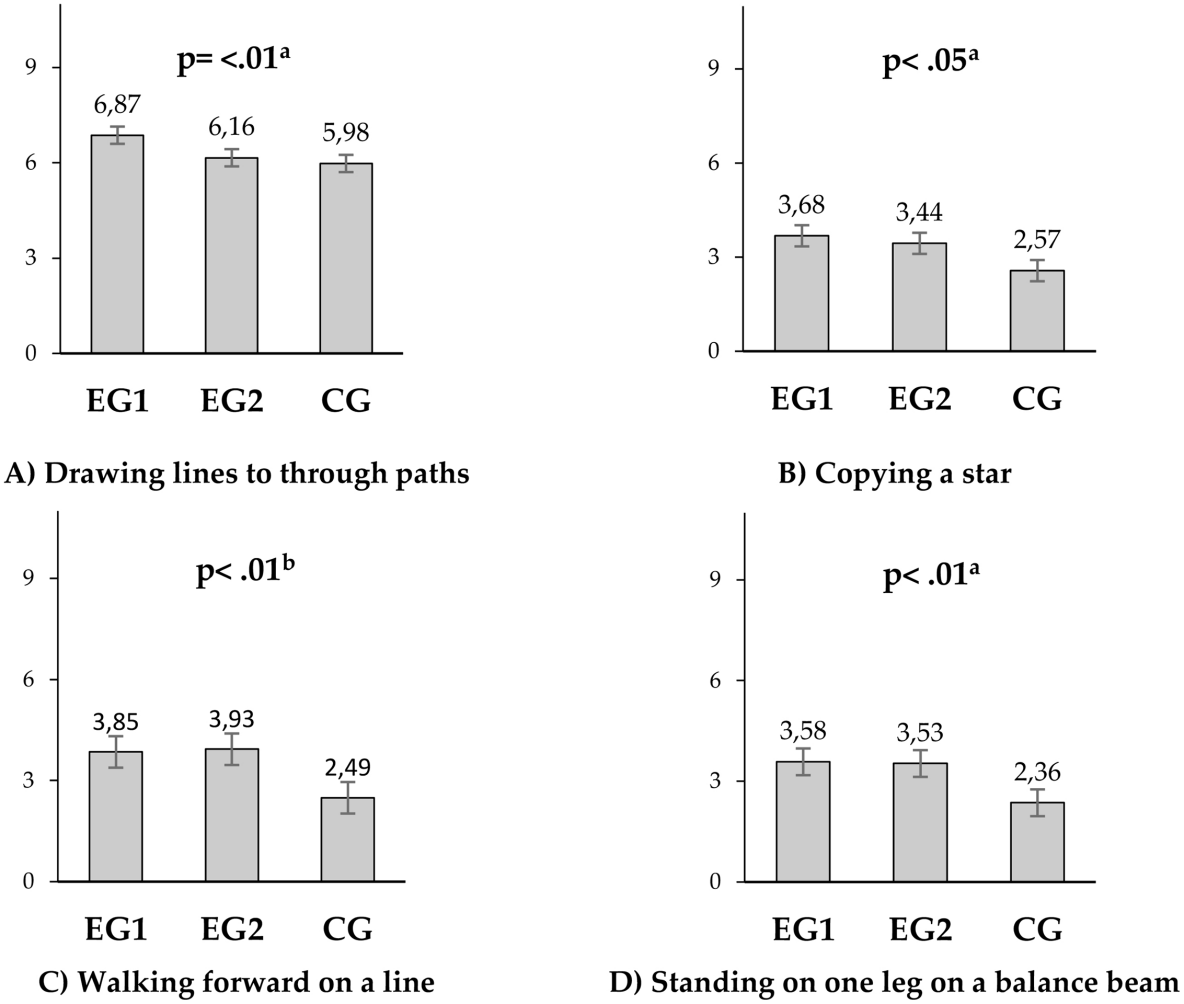
The effects of experimental programs (ANCOVA). EG1, experimental group 1 (2x/35 min per week, dance); EG2, experimental group 2 (3x/25 min per week, dance); CG, control group (2x/35 min per week, standard classes of physical education); The values are expressed as the Adjusted mean. P value refers to ANCOVA results a-group EG1, b-group EG2.

The comparisons and determination of real effects of dance programs with different frequencies (ANCOVA) between groups at the final measurement are illustrated in Fig 2. The results showed that statistically significant effects on motor competence were observed after the intervention with dance programs in both EG1 and EG2 groups, with similar improvements (based on adjusted means). The greatest effects in fine motor precision (Drawing lines through paths) and fine motor integration (Copying a star) were noted in the EG1 group (F = 7.67, p = 0.001, η2 = 0.17 and F = 4.02, p = 0.022, η2 = 0.10, respectively). Significant effects of dance programs with different frequencies were also observed in balance in both experimental groups, specifically in the One-legged balance on a balance beam test (F = 20.84, p = 0.001, η2 = 0.35) in the EG1 group, while a larger effect was observed in the Walking forward on a line in the EG2 group (F = 48.48, p = 0.001, η2 = 0.56) (Fig 2). Implemented dance programs with different frequencies did not lead to statistically significant effects in other areas assessing motor competence (manual dexterity, bilateral coordination, running speed and agility, upper body coordination, strength) (results not shown).

## Discussion

The results of the initial measurement had shown that there had been differences in certain tests for the assessment of motor competence between the groups. This may be explained, for instance, by the fact that children’s levels of physical activity had varied while they were not in kindergarten [24], by the respondents’ sex and body mass measures [52] temperamental traits like attention span activity and persistence [53] which may have affected the level of motor competence determined at the initial measurement.

Even if the scores of the minimum number of tests had decreased at the final measurement, the results nevertheless demonstrated that the implemented programs had a favor-able impact on motor competence. This can be explained by the adjustment period during which the body gets used to new movements and demanding activities, which can result in a temporary deterioration of certain motor skills before improvement occurs.

Nevertheless, the results of the research showed significant statistical effects of dance program interventions on motor competence in EG1 and EG2 compared to CG (Table 5, Fig 2). Improvements were similar in both groups, and the applied dance programs achieved greater effects on the tested parameters compared to the officially recommended regular exercise programme. These results align with the findings of other previous studies [29,35,54]. The most pronounced effects were observed in fine motor precision and fi-ne motor integration in the EG1 group, which practiced twice a week for 35 minutes each. The reason can be attributed to the larger volume of training in one session compared to the volume of training in EG2, which is more significant for the development of fine motor skills than shorter but more frequent activities. Similar results have been obtained in other studies [29,35,55]. Fine motor skills and manual dexterity involve the engagement of numerous small muscles and muscle groups, as well as good coordination between the eyes and hands, which are precisely contained in our experimental programs. Guo et al. [56]emphasize that this type of physical activity needs to be applied at the earliest age to improve fine motor skills and manual dexterity. The importance of fine motor development in later life has been explored through the analysis of predictors of academic success, and the results have shown that fine motor skills significantly influence future success in reading, mathematics, and science [57]. Enhancing children’s motor skills can have a lasting impact on promoting healthy lives and preventing non-communicable diseases [58], as well as a higher level of fitness [59].

In our study, it was found that a frequency of twice a week for 35 minutes of dance activity is sufficient to induce improvements in these abilities in preschool children.

Significant effects of dance programs with different frequencies were also found in balance in both experimental groups. Groups EG1 and EG2 had better results than CG in static balance (Standing on one leg on a balance beam—eyes open) and dynamic balance (Walking forward on a line with eyes open). That dance can improve dynamic balance in preschool-aged children was also determined by the study of Kapodistria & Chatzopoulos [36], emphasizing that dance programs consist of steps involving jumping, running, and standing on tiptoes, which requires activation of ankle muscles. These muscles play an important role in balance control [60], and a higher frequency of dance activity per week contributes to this improvement [54]. Kapodistria & Chatzopoulos [54] found improvement in static balance mediolateral direction in the experimental group but not in static balance anterior/posterior directions. The authors emphasize that the dances included in their study mainly consisted of lateral sequences of steps, which stimulate neuromuscular coordination involved in hip abduction and adduction, as well as ankle stabilization. This leads to stabilization in the mediolateral direction. However, in our study, besides dances containing lateral sequences of steps, dances involving forward-backward movements were also applied, which may explain the improvement in balance in EG2. The study by Chatzihidiroglou et al. [54] obtained similar results. Comparing their program [54], which lasted 8 weeks for 45 minutes, and the program implemented in the study by Wang [61], which lasted 6 weeks for 30 minutes of daily physical activity, it was found that the 8-week program contributed to improved balance, while the 6-week program did not achieve those results. In our research, positive results were recorded in both EG1 and EG2, with the note that in EG1, which had activities twice a week for 35 minutes each, better results were achieved in fine motor skills compared to EG2. Better results in EG1 in most parameters support the idea that it is possible to work with children twice a week but with a longer class duration, in our case, 35 minutes, over a period of 12 weeks. The study by Thomaidou et al. [29] achieved similar results in fine motor skills as in our study, but there was no improvement in any motor skill, which may be due to the duration of the program.

Our results (Table 5) show that there were large and moderate changes in other parameters for assessing motor competence in EG1 and EG2, but some were not statistically significant when post-hoc analyses were performed. Similar results were obtained in other studies with programs lasting 8 and 9 weeks [29,62]. Since the development of motor competence during certain ages has a high degree of inter-individual variation [63] and is not linear, with some children increasing, some remaining unchanged, and some even decreasing, and is generally marked by significant changes [64], the obtained results can also be explained by the assumption that the development of these parameters is not the same for all children. However, in the study by Anjos & Ferraro [65], whose dance program lasted seven months, twice a week for an hour, the EG group achieved better results in motor skills than the CG, indicating that for the improvement of certain motor competence parameters, the program must be of longer duration, while fine motor skills and balance can be improved in 12 weeks, as is the case with our study. In addition to dance activities, the study by Jaksic et al. [4] applied other activities that included natural movements with elements of athletics, exercises to improve physical fitness, elementary gymnastics, elementary games, and exercises to strengthen individual muscle groups. The program applied by the authors lasted 9 months and achieved improvements in both cognitive and motor skills in preschool children. This data indicates that a dance program certainly contributes to improving cognitive abilities, fine and gross motor skills in pre-school children, but it should be combined with other activities over a longer period of time.

Based on the stated results, the practical implication of the study involves the potential application of dance programs that include traditional and children’s dances as possible models for regular physical activity for preschool children in kindergartens. It is important to acknowledge that preschoolers’ immature neuromuscular systems can make learning complex rhythm patterns challenging. Nevertheless, the children demonstrated high levels of enjoyment during the sessions, which likely contributed to their full participation and minimal attrition. Our observational data on engagement aligns with findings from other feasibility studies of preschool dance and movement programs, which similarly report high enjoyment and adherence when activities are age-appropriate and engaging [66,67].

The study has some limitations. A weakness of the study is that we were unable to track children’s physical activity levels while at home, which could potentially influence the development of motor competence. It should also be noted the level of engagement in physical activities among this population during their time in daycare, as it has been found that their activities are predominantly sedentary [68,69]. Recent studies have reported that young children are sedentary for almost 50% of their time at childcare. We did not take into account the active involvement of educators in children’s play, as there is a connection between the personal engagement of educators in play and children’s physical activity, which can contribute to improving children’s motor competence [70].

More extensive studies lasting longer periods would be necessary to determine the effects more deeply. Future interventions should consider including formal measures of enjoyment and perceived competence, as well as gradually increasing the complexity of motor tasks,in order to optimize both safety and adherence amongpreschool children. The advantage of the research is that the results show that dance programs of varying frequencies lead to changes in children’s motor competence.

## Conclusion

The results suggest that dance programs implemented instead of the regular physical education program recommended by the Ministry of Education of the Republic of Serbia may yield greater effects, showing that 12-week dance programs included in the preschool curriculum may lead to significant improvements in fine motor skills and balance in pre-school-aged children. The results demonstrated that longer sessions, as in EG1, had more of an impact than shorter sessions held more frequently, as in the EG2 group. Preschool institutions may want to put into practice dance programs, either 2 sessions of 35 minutes each or 3 sessions of 25 minutes each per week, to increase motor competence, with the note that programs implemented with lower frequency but higher volume per session lead to greater improvements in motor competence. These results provide support for the inclusion of dance programs in preschool programs to enhance children’s motor skills.
